# A First Step towards a Comprehensive Approach to Harmonic Analysis of Synchronous Peripheral Volume Pulses: A Proof-of-Concept Study

**DOI:** 10.3390/jpm11121263

**Published:** 2021-12-01

**Authors:** Hsien-Tsai Wu, Bagus Haryadi, Jian-Jung Chen

**Affiliations:** 1 Department of Electrical Engineering, National Dong Hwa University, No. 1, Sec. 2, Da Hsueh Rd., Hualien 97401, Taiwan; hsientsaiwu@gmail.com (H.-T.W.); bagus.haryadi@fisika.uad.ac.id (B.H.); 2 Department of Physics, Universitas Ahmad Dahlan, Jendral A. Yani Street, Kragilan, Tamanan, Kec. Banguntapan, Bantul, Yogyakarta 55191, Indonesia; 3 Taichung Tzuchi Hospital, The Buddhist Tzuchi Medical Foundation, Taichung 42743, Taiwan; 4 School of Post-Baccalaureate Chinese Medicine, Tzu Chi University, Hualien 97002, Taiwan

**Keywords:** harmonic analysis, radial arterial waveform, peripheral volume pulse, digital volume pulse, photoplethysmography (PPG), discrete-time Fourier series (DTFS), averaged total pulse energy

## Abstract

The harmonic analysis (HA) of arterial radial pulses in humans has been widely investigated in recent years for clinical applications of traditional Chinese medicine. This study aimed at establishing the validity of carrying out HA on synchronous peripheral volume pulses for predicting diabetes-induced subtle changes in heart energy. In this study, 141 subjects (Group 1: 63 healthy elderly subjects; Group 2: 78 diabetic subjects) were enrolled at the same hospital. After routine blood sampling, all synchronous electrocardiogram (ECG) and photoplethysmography (PPG) measurements (i.e., at the six locations) were acquired in the morning. HA of synchronous peripheral volume pulses and radial pulse waves was performed and analyzed after a short period of an ensemble averaging process based on the R-wave peak location. This study utilized HA for the peripheral volume pulses and found that the averaged total pulse energy (i.e., the C_0_ of the DTFS) was identical in the same subject. A logistic regression model with C_0_ and a waist circumference variable showed a graded association with the risk of developing type 2 diabetes. The adjusted odds ratio for C_0_ and the waist circumference were 0.986 (95% confidence interval: 0.977, 0.994) and 1.130 (95% confidence interval: 1.045, 1.222), respectively. C_0_ also showed significant negative correlations with risk factors for type 2 diabetes mellitus, including glycosylated hemoglobin and fasting plasma glucose (r = −0.438, *p* < 0.001; r = −0.358, *p* < 0.001, respectively). This study established a new application of harmonic analysis in synchronous peripheral volume pulses for clinical applications. The findings showed that the C_0_ could be used as a prognostic indicator of a protective factor for predicting type 2 diabetes.

## 1. Introduction

There are several data decomposition methods in pulse wave analysis, of which harmonic analysis (HA) for discrete-time Fourier series (DTFS) [1,2,3], wavelet transformation [4], and ensemble empirical mode decomposition [5], among others, are efficient ways of viewing waveforms with regard to time/frequency or a nonlinear domain. The HA of arterial pressure pulses has standard protocols and reliability assessments, as well as being established in [6,7], for effective harmonic wave analyzers (TD01C, Mii-Ann Tech. Twain). In addition, as described in Matos et al.’s review [8] and Peng et al.’s report [9], the HA of radial arterial waveforms could make a significant contribution to traditional Chinese medicine. Professors Tsai et al. indicated that the harmonic characteristics of the radial artery in the wrist at different positions and different parameters were not identical, and recommended that any measurement location in future studies on the HA of pressure pulse diagnosis needs a more finely adjusted analysis [10]. More importantly, there have been further studies about the HA of radial arterial waveform (e.g., a kind of peripheral pressure pulse) addressing hypertension [11] and monitoring applied to coronary artery disease [12], type 2 diabetes mellitus [13,14,15,16], and the preservation of health [17]. On the other hand, a noninvasive wearable MEMS pressure sensor array was proposed in [18] for monitoring the arterial pulse waveform and heart rate, and the detection of atrial fibrillation. A number of similar reports on HA with a single channel pressure pulse signal have thus been addressed in the literature.

There is a vast range of photoplethysmography (PPG: one type of peripheral volume pulse) applications in healthcare, and there is a strong research focus on using PPG in wearable sensors [19]. An important previous study verified that the peripheral pressure pulse is related to the digital volume pulse (being one type of peripheral volume pulse); it can be obtained by measuring the infrared light transmission through finger PPG using a non-linear transfer function [20]. In their book [21], Professors Kyriacou and Allen discuss PPG with a focus on its applications in clinical physiological measurements, including clinical physiological monitoring, vascular assessment, and autonomic function, which are widely used in instrumentation, measurement protocols, and pulse wave analysis. However, there remain several problems for PPG volume pulses to overcome [22]. These include the instability of the volume pulses for PPG sensors. Therefore, a few studies have focused on the harmonic analysis (HA) of peripheral volume pulse waveforms (e.g., [23], which reported on only a single channel peripheral volume pulse but no clinical applications). We believe that wearable PPG and its applications are an increasing trend [21]. Therefore, the HA of the peripheral volume pulse in PPG has a great potential for the development of new solutions in the clinical application of traditional Chinese medicine.

Accordingly, our previous study [5] utilized radial arterial waveforms from the wrist and identified the diastolic peak from the fifth decomposed component through the ensemble empirical mode decomposition method, which was compared with the conventional digital volume pulse waveform [24,25] from the finger as an indicator of diabetic control in people who are older. To overcome the limitations of HA of the peripheral volume pulse waveform, the current study adopted synchronous whole-body peripheral volume pulse waveforms. We previously proposed a non-invasive six-channel electrocardiography (ECG)–PWV system [24,25] that could complete the calculations of six PWV values obtained from the bilateral earlobes, fingers, and toes within 30 min. Although the reliability of the system used in type 2 diabetes subjects and normal subjects was validated for the calculation of many human physiological parameters with PPG signals in our previous study (e.g., pulse wave velocity [24], multiscale entropy [24], and percussion entropy [26]), it had not been tested in subjects with HA computation. In another previous study [27], an animal model (i.e., a pig animal model) was anesthetized and attached to a medical monitoring device that records ECG, capnography, and PPG waveforms concurrently. The ultimate result was acoustic waveforms and time-registered data streams for heart and lung function. The recorded data came from sensors positioned in three different anatomical body locations (ear, leg, and tail). The study reported initial proof-of-concept large animal (porcine) experiments and a robust processing algorithm that demonstrates the feasibility of this approach.

There are a number of similar reports in the literature regarding HA with a single channel of pressure pulse signals; however, a novel application of the HA of synchronous multiple channels of peripheral volume pulses has not been seen before. This study focuses on creating a first step towards a comprehensive approach to the harmonic analysis of synchronous peripheral volume pulses. Specifically, synchronous ECG and PPG, as well as radial arterial waveforms, were measured at different locations in the body. Those PPG signals all belong to the peripheral volume pulse. The aims of this study were two-fold. First, we attempted to establish a feasibility assessment of the HA of synchronous peripheral volume pulses. Second, we validated the application of the HA of C_0_ and other incidence risk factors for type 2 diabetes mellitus prediction in older subjects. Subsequently, the current study needed to show that the averaged total pulse energies (i.e., C_0_ in discrete-time Fourier series (DTFS)) [1] decomposed from six synchronous peripheral volume pulses were identical or non-identical in the same subject.

A demonstration of the HA formula for the peripheral volume pulses and the statistical methods (e.g., Bland–Altman and binary logistic regression analysis) for the study are presented in Section 2. Subsequently, the values of the averaged total pulse energy are compared (identical or non-identical) for the six synchronous peripheral volume pulse signals in the same subject in Section 3. In addition, the agreement between the radial arterial waveform from the left wrist and the digital volume pulse from the left index finger on the averaged total pulse energy was assessed. Binary logistic regression analysis with a backward stepwise approach in SPSS for the incidence of risk factors for type 2 diabetes is also presented in Section 3. A summary of the research is presented, and the findings of the study are discussed and interpreted in Section 4. Finally, Section 5 concludes the manuscript.

## 2. Materials and Methods

### 2.1. Grouping of Middle-Aged Healthy and Diabetic Subjects

The study population consisted of 151 middle-aged subjects who underwent PPG and ECG examinations in the hospital. Subjects with or without type 2 diabetes and who were participating in the diabetes clinics were recruited (Table 1). A total of 10 participants were excluded due to a history of atherosclerosis-associated complications, including permanent pacemaker implantation, heart failure, coronary heart disease, and ischemic stroke, leaving 141 subjects remaining. In the study, diabetes mellitus was defined as a fasting glucose level higher than 126 mg/dL and/or a glycated hemoglobin (HbA1c) level greater than 6.5%.

### 2.2. Study Procedure

#### 2.2.1. Clinic Visit for Type 2 Diabetes

Anthropometric, demographic, and laboratory data for the analysis as well as medical history were obtained during the patients’ clinic visits for type 2 diabetes once over a period of three months. After the clinic visit, the test subjects were asked to provide blood samples. The total cholesterol, triglycerides, low-density and high-density lipoprotein cholesterol, fasting blood glucose, and glycosylated hemoglobin concentrations were obtained from blood samples after a 12-h fast. Caffeine-containing beverages and theophylline-containing drugs were forbidden for 12 h before each clinic visit.

#### 2.2.2. Data Measurement

After routine blood sampling, all the ECG and PPG measurements were carried out in the morning (08:30–10:30) in a test room. All the subjects completed a written informed consent form before the measurements were taken. Subsequently, the resting blood pressure was measured once from the left arm in a supine position using an oscillometric device (BP3AG1, Microlife, New Taipei, Taiwan). In this study, we adopted synchronous ear lobe, index finger, and second toe PPG signals, where noninvasive PPG sensors and ECG electrodes were applied to different locations of each subject for 30 min for data acquisition with a 500 Hz sampling rate. Moreover, to minimize latent erroneous readings from the PPG sensors arising from involuntary body movements of the test subjects and low environmental temperatures, possibly resulting in constriction of the peripheral vessels, all the subjects underwent blood sampling before data acquisition. All test subjects were allowed to relax in a supine position for 5 min in a quiet room with the temperature controlled at 26 ± 1 °C [24].

### 2.3. Harmonic Analysis of Synchronous Peripheral Volume Pulses

The synchronous analog signals (i.e., ECG and PPG) were then digitized with an analog-to-digital converter (i.e., USB-6009 DAQ, National Instruments, Austin, TX, USA) with a sampling frequency of 500 Hz; the digitized signals were stored on a personal computer for later data analysis.

#### 2.3.1. Radial Arterial Waveform and Digital Volume Pulse for the Same Subject

The radial arterial waveform from the left wrist in [5] and digital volume pulse from the left index finger [24] are both repetitive signals. However, one’s heart rate moderately changes between the beats of the human heart; thus, the radial arterial waveform and digital volume pulse beats show some similarity to the periodic waveform. Therefore, the radial arterial waveform and digital volume pulse could be regarded as quasi-periodic signals, which can be viewed as multiple interleaved stationary processes [28].

**Figure 1:**
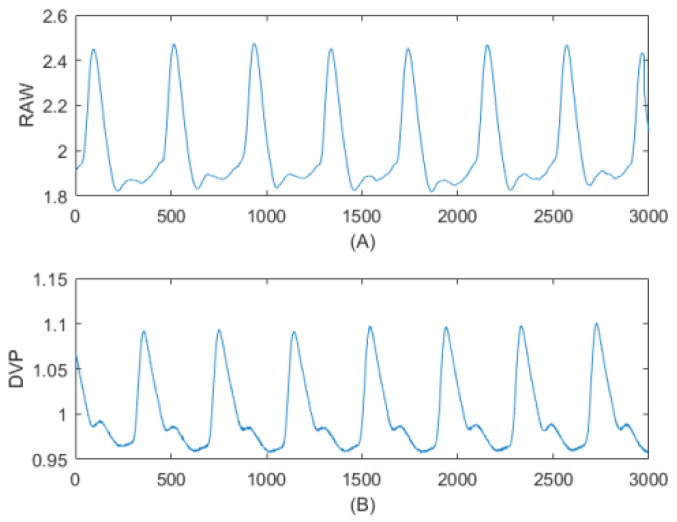
According to the quasi-periodic signal, (**A**) radial arterial waveform from the left wrist [5] and (**B**) digital volume pulse from the left index finger [24] could be regarded as cyclo-stationary, which is a signal having statistical properties that vary cyclically with time. Both waveforms consisted of 3000 consecutive points of sampled data over 6 s at a 500 Hz sampling frequency.

Like all continuous period signals, the radial arterial waveform and digital volume pulse (as shown in Figure 1), both through a digitized representation (i.e., sampled signal) using the analog-to-digital (A/D) converter, could be represented by a fundamental frequency sine wave and a collection of harmonics of that fundamental sine wave and summed together linearly (i.e., discrete-time Fourier series, DTFS) [28]. However, as the radial arterial waveform (a kind of pressure pulse signal) is suitable for HA as described in [6,7], the application of HA in the digital volume pulse (a kind of volume pulse signal) needs to be proved using a Bland–Altman plot for the assessment of agreement in the results.

#### 2.3.2. Data Acquisition and Harmonic Analysis in the Study

1. Data acquisition for peripheral volume pulses

**Figure 2:**
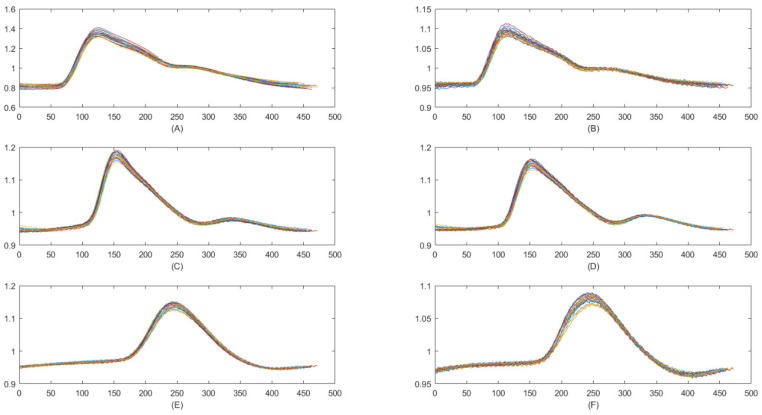
A total of 20 consecutive peripheral volume pulses measured from a six-channel electrocardiogram-based pulse wave velocity system (ECG-PWV) show different peripheral volume pulses from the (**A**) left ear lobe, (**B**) right ear lobe, (**C**) left index finger, (**D**) right index finger, (**E**) left second toe, and (**F**) right second toe, from one representative subject in Group 1.

A self-developed system (i.e., a noninvasive ECG-PWV-based system [24]) was provided to acquire successive data points sampled over 30 min for the ECG signals and peripheral volume pulses at a 500 Hz sampling rate. In this study, we adopted synchronous ear lobe, index finger, and second toe PPG signals (Figure 2), and a lead II ECG signal for determining the precise periods of the peripheral volume pulse.

2. Harmonic analysis for peripheral volume pulses

According to the concept of quasi-periodic signals in [29], the peripheral volume pulse could be viewed as multiple interleaved stationary processes and represented with discrete-time Fourier series (DTFS). Although PPG pulses sampled successively for 30 min from the six locations were acquired, only Np period signals (Np+1 heart beats) from the second minute were used for the DTFS computation in this study, because most of the tested subjects were awake in that time interval and the PPG signals were more stable. Subsequently, the PPG signals were adopted for DTFS computation.

For example, we retrieved 100 consecutive periods (e.g., Np = 100) of peripheral volume pulses according to the location of the R peaks of the ECG, shown as {x[n]} = {x[1], x[2], x[3], …, x[N]} (e.g., N sampled peripheral volume pulse data), with 100 different periods, T_1_, T_2_, …, T_100_. If we assume that T_1_ = T_2_ = … = T_100_ = 1 s, then N = 50,000, with a sampling rate of 500 Hz. In that case, {x[n]} is a complete period discrete signal, and thus, {x[n]} = {x[n + k × 500]}, k = 0, 1, 2, …, 99, which can be decomposed as follows:

(1) $$\{x[n]\}=\frac{1}{500}\sum_{k=0}^{500-1}X\left(k\right)e^{i\frac{2\pi }{500}\mathrm{kn}},\;n=1,\;2,\;\ldots ,\;50,000,$$

where {X(k), k = 0, 1, 2, …, 10} is the first 11 coefficients of the DTFS of x[n], which is as suggested in [6,7]; it can also be represented as

(2) $$\{X(k)\}=\sum_{n=0}^{500-1}x\left[n\right]e^{-i\frac{2\pi }{500}\mathrm{kn}},\;\;k=0,\;1,\;2,\;\ldots ,\;10,$$

whereas the peripheral volume pulse is regarded as the quasi-periodic signal; all the T_i_ (T_1_, T_2_, …, T_Np_) are similar but not identical. All the peripheral volume pulse signals were separated sequentially by the R peaks from the foot point of the peripheral volume pulse to the next foot point. In addition, the DTFS process is explained as follows:

Step 1. A total of Np periods of the peripheral volume pulse was determined precisely, using the ensemble averaging process based on the R-wave peak location.

Step 2. The Fourier series coefficients for each period of the peripheral volume pulse were found:

(3) $$\{X(k,j)\}=\sum_{n=0}^{500-1}x_{j}\left[n\right]e^{-i\frac{2\pi }{500}\mathrm{kn}},\;\;k=0,\;1,\;2,\;\ldots ,\;10,\;$$

where x_j_[n] indicates the jth-period pulse, and k indicates the kth harmonic component of the 100 periods of the peripheral volume pulse.

Step 3. The averaging amplitude value of each period of the peripheral volume pulse was calculated:

(4) $$\left|X\left(k,j\right)\right|=\frac{1}{500}\sum_{j}{\left|x_{j}\left[n\right]\right|}_{\;},\;\;j=1,\;2,\;\ldots ,\;\mathrm{Np}.\;$$

The mean of j vectors of {**X**(k,j)} is a vector with j means.

Step 4. The Fourier series coefficients for each period were normalized: {X(k,j)}/|X(k,j)|, j = 1, 2, 3, …, Np. The kth row of the period j of {**X**(k,j)} must be divided by the absolute mean of {**X**(k,j)} of the period jth.

Step 5. The representative coefficients of the harmonic component was found:

(5) $$C_{k}=\frac{1}{100}\sum_{j}\frac{\left\{X\left(k,j\right)\right\}}{\left|X\left(k,j\right)\right|},\;k=0,\;1,\;2,\;\ldots ,\;10;\;j=1,\;2,\;\ldots ,\;\mathrm{Np}.$$

It is worth mentioning that C_0_ in (5), the driving force (energy) generated by the heart, was defined as the averaged total pulse energy of the averaged pulse waveforms for the arterial radial pulses in [1]. The other coefficients of the harmonic component (e.g., C_1_–C_10_) could reflect the matching condition between the heart rate and the natural frequencies of the arterial system [1]. Finally, in Step 5, the normalized Fourier coefficient was determined as the mean of the normalized Fourier coefficients calculated over the included periods.

### 2.4. Statistical Methods for the Study

Regarding the statistical analysis and logistic regression, a statistical software package (i.e., Statistical Package for the Social Sciences, Version 14.0, SPSS Inc., and Chicago, IL, USA) was utilized for verification. The signal analysis package used was Matlab 2016a (MathWorks Inc., Natick, MA, USA). The functions “dtfs” and “blandaltman” in Matlab were developed for the computation of the coefficients of the harmonic component in Equations (1)–(5) and Bland–Altman plot later, respectively.

#### 2.4.1. Bland–Altman Analysis

The study tested 33 elderly non-diabetic subjects, who underwent radial pulse wave measurements followed by 6 synchronous peripheral volume pulse measurements during a health examination program for agreement assessment. The HA of the radial pulse wave was shown to be a feasible and reliable method with which to assess the hemodynamic characteristics in healthy humans in [6,7]. The reliability assessment for the application of HA in the digital volume pulse was evaluated using the Bland–Altman analysis in this study. Thus, it was necessary to check the agreement between the radial arterial waveform (i.e., measured from an air-pressure-sensing system [5]) and digital volume pulse (i.e., measured using a noninvasive ECG-PWV-based system [24]) on C_0_ (i.e., averaged total pulse energy) before the volume pulse was used for DTFS computations.

#### 2.4.2. Statistical Analysis and Logistic Regression

For the comparison between coefficients (C_0_–C_10_) of the DTFS for the digital volume pulse signals from Group 1 (i.e., healthy elderly subjects) and Group 2 (i.e., diabetic patients), an independent t-test was used, with *p* < 0.05 regarded as statistically significant. The binary logistic regression model not only offers a statistical method for modeling a binary outcome, which takes values of 1 or 0, but can also be predicted by the level of one or more risk factors [30]. In this study, the fitted logistic regression model was used to estimate the probability of the presence or absence of type 2 diabetes and to find all the possible risk factors that influence the probability of the dichotomous outcome. The results of the logistic regression were also presented in terms of the odds of the outcome. The fit of the resulting model can be assessed using the Hosmer–Lemeshow test, R^2^ for the logistic regression, and the overall percentage in classification. Without using the information of the blood samples, we focused on the parameters that were easily obtained at home (i.e., age, waist circumference, body mass index, systolic blood pressure, diastolic blood pressure, pulse pressure, and human physiology parameters) for the prediction of risk factors of type 2 diabetes.

## 3. Results

In this study, six synchronous peripheral volume pulse signals were obtained from a six-channel ECG-PWV system, and the synchronous peripheral volume pulse signals were then decomposed with DTFS and analyzed, with the first eleven coefficients of the harmonic terms of the synchronous peripheral volume pulse signals (i.e., C_0_–C_10_ in DTFS). We found that the values of C_0_ were the same for the six synchronous peripheral volume pulse signals in the same subject, as described in Section 3.1. Subsequently, we assessed the agreement between the radial arterial waveform (i.e., measured using an air-pressure-sensing system [5]) and digital volume pulse for the same subjects on C_0_ (i.e., averaged total pulse energy) using a Bland–Altman plot in Section 3.2. A sufficiently smaller ensembled averaging number for the C_0_ computation, due to a shorter CPU time, was chosen, as described in Section 3.3. The reliability of C_0_ for differentiating type 2 diabetic patients from elderly subjects was then verified, as described in Section 3.4. Finally, a binary logistic regression model was fitted to establish whether C_0_ was a protective factor for type 2 diabetes mellitus in elderly subjects, as described in Section 3.5.

### 3.1. C_0_ from Synchronous Peripheral Volume Pulse Signals

**Figure 3:**
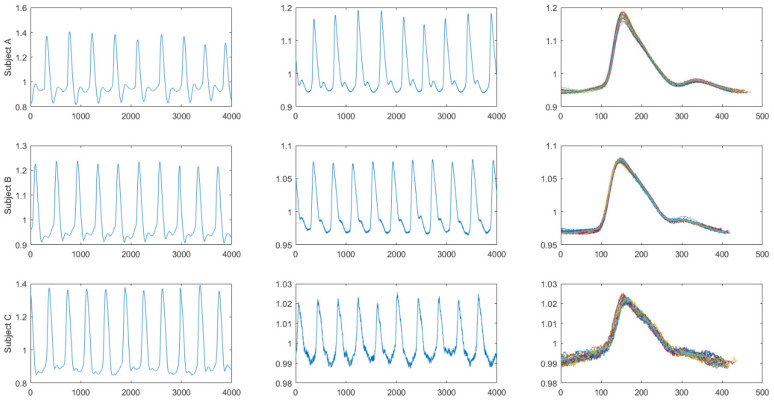
Variation in digital volume pulse from Subject A (healthy elderly subject), Subject B (healthy elderly subject), and Subject C (patient with type 2 diabetes). Left column: single left radial pulse from each subject. Middle column: single left index finger digital volume pulse from each subject. Right column: A total of 20-cycle overlapping index finger digital volume pulses from each subject, showing the widest variation in waveforms in Subject C.

Figure 3 illustrates the radial pulses and digital volume pulses of Subject A (a healthy elderly subject), Subject B (a healthy elderly subject), and the type 2 diabetes patient, Subject C, which appeared similar to the period signals. In qualitative research approaches, digital volume pulses can adopt DTFS analysis followed by radial pulses. The right column in Figure 3 shows the widest variation in waveforms in Subject C in 20 overlapping index finger digital volume pulses. Moreover, the periods of the digital volume pulse for the three subjects were determined precisely by using the ensemble averaging process based on the R-wave peak location, even for the diabetic subject.

Table 2 shows the first three coefficients (C_0_, C_1_, and C_2_) of the DTFS of the synchronous peripheral volume pulse, measured from six measured locations (i.e., the left ear, right ear, left index finger, right index finger, left index toe, and right index toe), from healthy elderly subjects A and B (i.e., in Group 1) and a diabetic subject C (i.e., in Group 2). The ensemble averaging number was set as 100. It is interesting that the values of C_0_ (i.e., averaged total pulse energy) were all the same for the peripheral volume pulse signals measured from different locations for the same subject, whereas C_1_ and C_2_ were not identical in the same subject.

### 3.2. Assessment of Agreement between Radial Arterial Waveform and Digital Volume Pulse on C_0_

**Figure 4:**
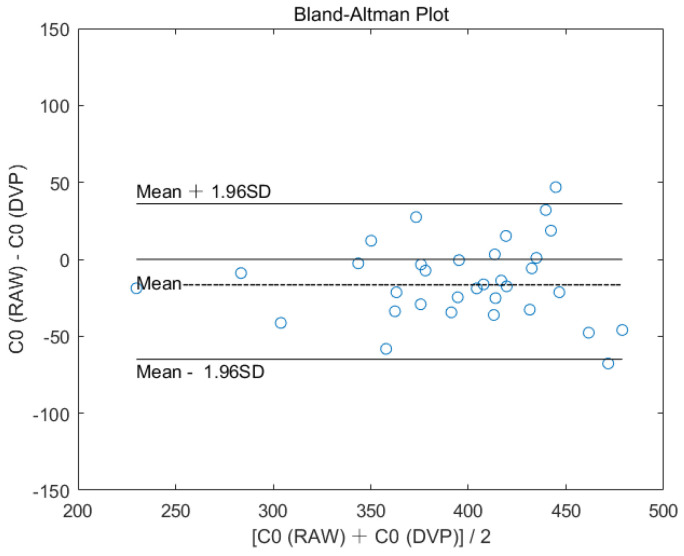
Bland–Altman plot showing good agreement between radial arterial waveform (i.e., measured from an air-pressure-sensing system) and digital volume pulse (i.e., measured from a six-channel ECG-PWV system) on C_0_ for 33 elderly non-diabetic subjects.

This agreement study tested 33 elderly non-diabetic subjects who accepted radial pulse wave measurements followed by six synchronous peripheral volume pulse measurements during a health examination program at the same hospital. Figure 4 shows the Bland–Altman plot of the averaged total pulse energy (i.e., C_0_) of the DTFS using the two pulse signals (e.g., C_0_ (RAW) vs. C_0_ (DVP)), with good agreement under both numbers of the ensembled averaging set as 100.

### 3.3. Choosing A Sufficient Number for Ensembled Averaging for C_0_ Computation

#### 3.3.1. Coordination for CPU Time vs. Ensembled Averaging Number

**Figure 5:**
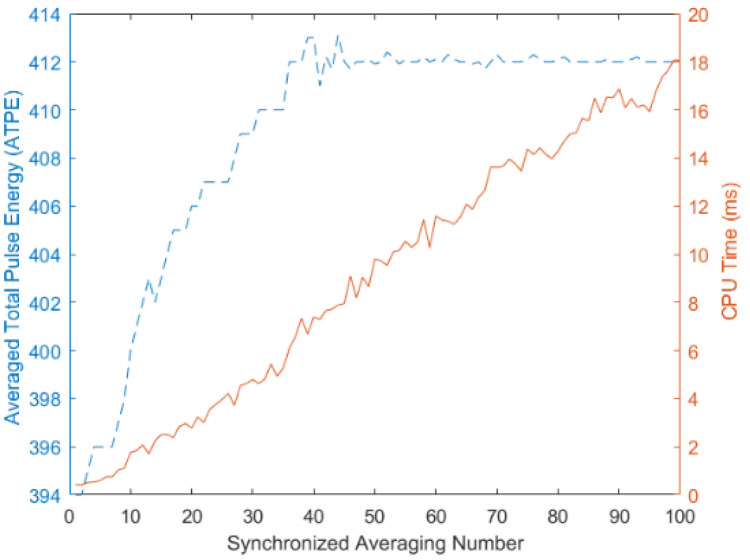
A double *y*-axis graph: Variation of the C_0_ values under different period numbers for ensemble averaging on the left *y*-axis. The secondary vertical axis for CPU time (ms) is added on the right for Subject A.

According to the DTFS representation, the more the harmonic terms existed, the more accurate the approach to the original digital volume pulse. It is well known that the computation load increases when more harmonic terms exist. Therefore, 12-s radial arterial waveforms were adopted for DTFS computation in [6,7]. In our study, Figure 5 shows that 20 cycles (i.e., more than 12 s) were a sufficient number for ensemble averaging for C_0_ computation in DTFS for Subject A (in Step 1 and Equations (3)–(5)). Figure 5 indicates (1) the C_0_ curve as greatly increasing, far more than the CPU time, and (2) the larger period number for the ensemble averaging for the C_0_ computation needed a longer CPU time (e.g., 20-period ensemble averaging vs. 2.78 ms, and 100-period ensemble averaging vs. 18.06 ms, with C_0_ = 405.8 and C_0_ = 411.8, respectively).

#### 3.3.2. Reproducibility of C_0_ Using Digital Volume Pulse Measured from Left Index Finger

The intra-class coefficient of the correlation between the two separate measurements of C_0_ was high (r = 0.947, *p* < 0.001). There were no significant differences between the two measurements (392.71 ± 69.95 vs. 394.75 ± 71.98, *p* = 0.528; mean difference, 2.03 ± 18.31). The reproducibility of the C_0_ study was tested in 33 elderly non-diabetic subjects who were chosen randomly from Group 1. The number for ensembled averaging was set as 20.

### 3.4. Reliability of C_0_ in Differentiating Type 2 Diabetic Patients

#### 3.4.1. C_0_ Is associated with Type 2 Diabetics

Table 3 illustrates the results of the comparison of the DTFS coefficients (C_0_–C_10_) of 20-cycle ensembled averaging digital volume pulse waveforms between the two groups of test subjects. There was a notably significant difference in C_0_ between the two groups (*p* < 0.001). For the other coefficients (C_1_–C_10_), there were no notably significant differences between the two groups (all *p* > 0.05).

#### 3.4.2. Correlation of Type 2 Diabetic Risk Factors with C_0_

The parameter C_0_ also showed significant negative correlations with other risk factors for type 2 diabetes mellitus, including glycosylated hemoglobin and fasting plasma glucose (r = −0.438, *p* < 0.001; r = −0.358, *p* < 0.001, respectively), waist circumference (r = −0.349, *p* < 0.001), body weight (r = −0.295, *p* < 0.05), and body mass index (r = −0.246, *p* = 0.03).

The following section will address the associations between age, waist circumference, body mass index, systolic blood pressure, diastolic blood pressure, and pulse pressure; C_0_ (i.e., risk factors) was also examined regarding the probability of the occurrence of type 2 diabetes mellitus using the binary logistic regression model.

### 3.5. Discrimination of Binary Logistic Regression Model Using SPSS

The binary logistic regression model is a type of predictive modeling that can be used in a study when the response variable is binary, meaning that there are only two possible outcomes, such as healthy or type 2 diabetes mellitus. The binary logistic regression analysis with a backward stepwise approach in SPSS for incidence risk factors for diabetes mellitus is shown in Table 4. As continuous variables, C_0_ and waist circumference were statistically significantly associated with the risk of developing diabetes mellitus. The relative risk of diabetes mellitus for the C_0_ was 0.986 (*p* = 0.001), whereas the relative risk of diabetes mellitus for the elderly subjects according to waist circumference was 1.131 (*p* = 0.002). A *p*-value of <0.05 was noted as statistically significant for the test. The overall goodness of fit of the model was then verified (Hosmer–Lemeshow test: χ^2^ = 11.19; degrees of freedom = 8, *p* = 0.191; R^2^ for logistic regression: Cox–Snell R^2^ = 0.376 and Nagelkerke R^2^ = 0.504; overall percentage in classification table = 80.4%). The values of R^2^ (i.e., 0.376 and 0.504) and the overall percentage (i.e., 80.4% > 60%) showed the fitted model to be useful for predicting diabetes mellitus in elderly subjects in this study.

As shown in Table 4, the fitted logistic model is

logit (P) = ln [P/(1 − P)] = −6.573 + 0.122 × waist circumference − 0.015 × C_0_. (6)

The P in Equation (6) shows the probability of type 2 diabetes mellitus (i.e., P ≥ 0.5 will be classified as type 2 diabetes mellitus; P < 0.5 will be classified as healthy elders). The operator ln is defined as the natural logarithm operation. The crude odds ratio for C_0_ was 0.981. After controlling for age, body weight, body mass index, systolic blood pressure, and diastolic blood pressure, the adjusted odds ratio for C_0_ was 0.986 (95% confidence interval: (0.977, 0.994)). This indicates that, for the given values of C_0_, the odds of developing diabetes mellitus are multiplied by 0.986 for every 1% increase in C_0_. A larger C_0_ value means less chance of developing diabetes mellitus.

## 4. Discussion

In the current study, we proposed the first eleven coefficients (C_0_–C_10_) of the DTFS of the synchronous peripheral volume pulse, which were measured from six measured locations (i.e., the left ear, right ear, left index finger, right index finger, left index toe, and right index toe) (Figure 3 and Table 2). Accordingly, we utilized HA for six synchronous peripheral volume pulses, and found that the averaged total pulse energy (i.e., the C_0_ of the DTFS) was the same for the same subject (Table 2). We thus recommend its clinical application for type 2 diabetes (Table 3). In summary, HA could confirm that diabetic patients had smaller C_0_ values for any one of the six synchronous peripheral volume pulse waveforms measured from the ECG-PWV system [24,25]. After controlling for age, body weight, body mass index, systolic blood pressure, and diastolic blood pressure, the adjusted OR for C_0_ was 0.986 (95% CI: 0.977, 0.994) in Equation (6). This indicates that, for the given values of C_0_, the odds of developing diabetes mellitus are multiplied by 0.986 for every 1% increase in C_0_. A larger C_0_ value means less chance of developing type 2 diabetes mellitus (Table 4). C_0_ also showed a significant negative correlation with two risk factors for type 2 diabetes mellitus, including glycosylated hemoglobin and fasting plasma glucose (r = −0.438, *p* < 0.001; r = −0.358, *p* < 0.001, respectively), as described in Section 3.4.2. According to C_0_ (the driving force generated by the heart) in Equation (5), and defined as the averaged total pulse energy of the averaged pulse waveforms for arterial radial pulses in [1], the simultaneous peripheral volume pulses from ear PPG, finger PPG, and toe PPG sensors could be decomposed for many harmonic components via DTFS and lead to the same C_0_ (harmonic zero) for the same subject in the current study, which is consistent with the hypothesis in [6,7] with a single channel of pressure pulse signals. Our study serves as a proof of concept in traditional Chinese medicine (especially in the classic of Chinese medicine named “*Yellow Emperor’s Internal Classic*”) that the heart, as a monarch, governs the blood [1], controls the blood vessels [31], and governs the whole body’s continuous activity [32]. On the other hand, unlike the hypothesis in [6,7], C_1_–C_10_ could not be identical for the six simultaneous peripheral volume pulse signals for the same subject (Table 2), consistent with the findings in [10]. It is reasonable to infer that the natural frequencies of the arterial system would be different for the six peripheral volume pulse signals, which were measured at six different locations [1]. The findings in this study imply that the HA of the synchronous peripheral volume pulse for assessing C_0_ is feasible, but C_1_–C_10_ must be further tested.

A previous study showed that the peripheral pressure pulse is related to the digital volume pulse [21]. In this study, good agreement between the radial arterial waveform from the wrist, the digital volume pulse from the finger, and the average total pulse energy (i.e., C_0_) of DTFS can be observed in Figure 4. It is also proposed that 20 cycles for a single channel of pressure pulse signals [6,7] is a sufficient number for the ensemble averaging process for the C_0_ computation in DTFS (Figure 5). To achieve stable PPG waveforms, the ensemble average number would be taken from 20 to 100 cycles of steady waveform recordings. In addition, we established a five-step HA computation formula for the peripheral volume pulse, described in Section 2.3.2. Hence, the current study obtained results for type 2 diabetes mellitus [14,15,16,17]. It has been reported that the fourth harmonic component of the radial pulse wave is important for the risk assessment of macrovascular and microvascular events in patients with type 2 diabetes [14], cardiovascular risk [15], and arterial blood pressure waveforms [16], with different radial pulse wave harmonic indices, silent coronary artery disease, and adverse cardiac events [17]. Therefore, our findings for synchronous peripheral volume pulses are consistent with the findings in [14,15,16,17], in which the radial pulse wave was adopted with different harmonic coefficients.

Unlike the stable peripheral pressure pulse (e.g., the radial arterial waveform), the peripheral volume pulse (e.g., the digital volume pulse), due to its advantages as a non-invasive, inexpensive, and convenient diagnostic tool, is suitable for wearable applications [21,22,24]. For example, we may place PPG sensors in eardrops, finger cots, and toe sleeves for peripheral volume pulse acquisition from the ear, index finger, and toe, respectively. In addition, novel algorithms could extract information from the unstable PPG waveforms in potential applications (e.g., AI techniques), which would provide assistance in the HA of the synchronous peripheral volume pulse. For the issue regarding the removal of PPG motion artifacts, some review studies [33,34] have presented several compensation techniques, such as independent component analysis, adaptive noise cancellation, and methods with/without learning-based algorithms for the design of wearable devices. According to a vast range of PPG applications in healthcare [21], with a strong focus on the contribution of PPG in wearable sensors and PPG for cardiovascular-related disease assessment, there are two key points: (1) optical components enabling the extreme miniaturization of such sensors; and (2) the comprehensive coverage of PPG signal analysis techniques, including machine learning and artificial intelligence. A recent study focused on the HA of peripheral volume pulse waveforms [22], revealing a loss of high-frequency harmonic components in the peripheral pulse for coronary artery disease subjects. However, this result was not consistent with our study. There was a notably significant difference in C_0_ between the two groups (*p* < 0.001), while for the other coefficients (C_1_–C_10_) (e.g., high-frequency harmonic components), there were no notably significant differences (all *p* > 0.05) (Table 3) in the current study. Following the inference of the difference compared with [22] in the above results, the digital volume pulse in our study was determined precisely during the period using the ensemble averaging process based on the R-wave peak location before HA computation, which is the most likely reason for the difference. On the other hand, 20-cycle digital volume pulse waveforms with ensemble averaging were adopted in Table 3 for DTFS coefficient (C_0_–C_10_) computation. More importantly, HA [6,7], wavelet transformation [4,35] (both linear models), and ensemble empirical mode decomposition [5] (a non-linear model) are three popular signal decomposition methods for biomedical signal analysis, which result in orthogonal harmonic components, orthogonal wavelet details, and orthogonal intrinsic mode function outputs, respectively. A reasonable inference would be that more stable and shorter peripheral volume pulses suggest significant improvements in HA computation for assessing the averaged total pulse energy in elderly and diabetic subjects, which is a potentially important issue when using an ensemble averaging process based on the R-wave peak location (as in Step 1 in Section 2.3.2).

This study has some limitations. The subjects in the two groups (not a wide-ranging clinical study; thus, the number of participants was limited) were not age-controlled for an unbiased analysis. Therefore, there was a wide gap between the healthy group and the diabetes group, which was beneficial for separating C_0_–C_10_. Second, as the pulse signal in a short period of time can be treated as a periodic signal due to its small non-linearity, the discrete-time Fourier series should be considered a powerful pulse analysis method. Third, there are a number of similar reports in the literature regarding HA with a single channel of pressure pulse signals. This study was a proof-of-concept study of the HA of synchronous multiple channels of peripheral volume pulses. Finally, Stata and R packages were not adopted in the study, and are other statistical software packages that could be explored.

## 5. Conclusions

The current study established a first step towards a comprehensive approach via a proof-of-concept study of the harmonic analysis of synchronous peripheral volume pulses. Our findings not only highlighted that the averaged total pulse energies (i.e., the C_0_ of the DTFS) decomposed from six synchronous peripheral volume pulses were identical in all six cases, but also recommend the energy’s possible clinical use as a prognostic indicator of protective factors for predicting type 2 diabetes. One important future direction for these results will be contributing to the prediction of other diseases using either coefficients (i.e., the C*_i_* of the DTFS) and/or variation coefficients of the *i*th harmonic amplitude (i.e., the C*_i_*CV of the DTFS) in a clinical setting.

## Figures and Tables

**Table 1:** Anthropometric and serum biochemical parameters from Group 1—healthy elderly subjects, and Group 2—diabetic subjects.

Parameter	Group 1	Group 2	*p*-Values
	Mean ± SD or N (%)	Mean ± SD or N (%)	
Gender (male/female)	63 (29/34)	78 (44/34)	N/A
Age, year	54.59 ± 10.06	63.54 ± 8.33 **	<0.001
Body height, cm	162.58 ± 8.19	162.45 ± 8.54	0.927
Body weight, kg	64.32 ± 11.29	71.07 ± 11.11 *	0.001
WC, cm	83.25 ± 10.85	93.87 ± 9.78 **	<0.001
BMI, kg/m^2^	24.28 ± 3.68	26.88 ± 3.81 **	<0.001
SBP, mmHg	120.89 ± 14.58	123.99 ± 23.38	0.361
DBP, mmHg	74.86 ± 9.44	74.35 ± 13.77	0.802
PP, mmHg	46.03 ± 11.75	49.88 ± 15.51	0.105
LDL, mg/dL	124.95 ± 41.12	120.87 ± 38.22	0.543
Cholesterol, mg/dL	184.52 ± 66.30	185.28 ± 47.23	0.937
HbA1c, %	5.83 ± 0.35	8.35 ± 1.77 **	<0.001
FPG, mg/dL	99.48 ± 16.42	161.83 ± 35.71 **	<0.001

The total final number of test subjects was 141. Group 1, healthy elderly subjects; Group 2, diabetic subjects. WC, waist circumference; BMI, body mass index; SBP, systolic blood pressure; DBP, diastolic blood pressure; PP, pulse pressure; LDL, low-density lipoprotein cholesterol; HbA1c, glycosylated hemoglobin; FPG, fasting plasma glucose. ** *p* < 0.001, * *p* < 0.05, Group 1 vs. Group 2. The *p* values of the parameter less than 0.05 and 0.001 are regarded as statistically significant between the two groups.

**Table 2:** Three coefficients (C_0_, C_1_, and C_2_) of discrete-time Fourier series from Subjects A and B in Group 1—healthy elderly subjects, and Subject C in Group 2—diabetic elderly subjects.

Location for PVP Measurement	Subject A			Subject B			Subject C		
	C_0_	C_1_	C_2_	C_0_	C_1_	C_2_	C_0_	C_1_	C_2_
Left ear	429.5	32.3	21.3	410.8	13.7	5.8	403.2	40.5	14.8
Right ear	429.5	10.6	5.8	410.8	3.2	1.4	403.2	6.7	2.3
Left index finger	429.5	14.8	10.4	410.8	8.2	4.6	403.2	2.2	1.0
Right index finger	429.5	13.1	10.0	410.8	10.0	5.8	403.2	2.3	1.2
Left index toe	429.5	4.8	2.9	410.8	13.6	8.1	403.2	12.6	7.8
Right index toe	429.5	8.6	4.8	410.8	18.9	10.1	403.2	5.4	3.4

Subjects A (age: 35 years) and B (age: 52 years) were two healthy subjects, whereas Subject C (age: 42 years) was a diabetic patient. PVP, peripheral volume pulse recorded simply and noninvasively by photoplethysmography in six locations [24] in this study. C*_i_*: the *i*th Fourier series coefficient of 100-cycle ensembled averaging digital volume pulse waveforms. C_0_ = the averaged total pulse energy [1] of the 100-cycle digital volume pulse waveforms with ensemble averaging.

**Table 3:** Coefficients (C_0_–C_10_) of discrete-time Fourier series for the digital volume pulse signals from Group 1—healthy elderly subjects—and Group 2—diabetic patients.

Coefficient	Group 1	Group 2	*p*-Values
	Mean ± SD	Mean ± SD	
C_0_	417.62 ± 44.80	363.05 ± 60.93 **	<0.001
C_1_	8.53 ± 5.47	8.15 ± 6.19	0.707
C_2_	3.55 ± 2.43	3.30 ± 2.41	0.545
C_3_	1.62 ± 1.12	1.46 ± 1.20	0.426
C_4_	0.86 ± 0.69	0.80 ± 0.64	0.568
C_5_	0.76 ± 0.56	0.68 ± 0.62	0.438
C_6_	0.45 ± 0.35	0.41 ± 0.44	0.532
C_7_	0.25 ± 0.23	0.25 ± 0.26	0.909
C_8_	0.18 ± 0.16	0.18 ± 0.21	0.987
C_9_	0.12 ± 0.10	0.13 ± 0.18	0.578
C_10_	0.07 ± 0.06	0.09 ± 0.12	0.138

The total number of test subjects was 141. Group 1—healthy elderly subjects; Group 2—diabetic subjects. ** *p* < 0.001, Group 1 vs. Group 2. C*_i_*: the *i*th Fourier series coefficient of digital volume pulse waveforms with 20-cycle ensemble averaging; C_0_ = averaged total pulse energy of the digital volume pulse waveforms with ensemble averaging. The ensemble averaging number was set as 20.

**Table 4:** Incident risk analysis of type 2 diabetics with two parameters.

Parameter	Coef.	Sign.	Exp(B)	95% CI for OR
C_0_	−0.015	0.001	0.986	0.977–0.994
WC	0.122	0.002	1.130	1.045–1.222
Constant	−6.573	0.046	0.001	–

Coef. = regression coefficients of the fitted model; Sign. = *p* value, *p* < 0.05 represents a model where the independent variable is statistically significant; Exp(B) = odds ratio (OR); CI: confidence interval; C_0_ = the averaged total pulse energy of digital volume pulse waveforms with 20-cycle ensemble averaging. WC = waist circumference. A backward stepwise approach for logistic regression analysis with a set likelihood ratio was adopted in SPSS. A *p*-value < 0.05 was considered statistically significant for the test parameter.

## Data Availability

Not applicable.

## Abbreviations

BMI: Body Mass Index; CI: Confidence Interval; DBP: Diastolic Blood Pressure; DTFS: Discrete-Time Fourier Series; ECG: Electrocardiography; FPG: Fasting Plasma Glucose; HA: Harmonic Analysis; HbA1c: Glycosylated hemoglobin; HDL: High-Density Lipoprotein cholesterol; LDL: Low-Density Lipoprotein cholesterol; PPG: Photoplethysmography; PWV: Pulse Wave Velocity; SBP: Systolic Blood Pressure; SPSS: Statistical Package for the Social Sciences; TG: Triglyceride; WC: Waist Circumference.
